# MET Signaling Pathways, Resistance Mechanisms, and Opportunities for Target Therapies

**DOI:** 10.3390/ijms232213898

**Published:** 2022-11-11

**Authors:** Solange Rivas, Arnaldo Marín, Suraj Samtani, Evelin González-Feliú, Ricardo Armisén

**Affiliations:** 1 Centro de Genética y Genómica, Instituto de Ciencias e Innovación en Medicina, Facultad de Medicina Clínica Alemana, Universidad del Desarrollo, Santiago 7550000, Chile; 2 Departamento de Oncología Básico Clínica, Facultad de Medicina, Universidad de Chile, Santiago 8380000, Chile; 3 Departamento de Oncología Médica, Clínica Las Condes, Santiago 7550000, Chile; 4 Hospital Félix Bulnes, Santiago 9080000, Chile

**Keywords:** precision medicine, NSCLC, target therapies, resistance mutations, driver mutations, actionable mutations

## Abstract

The *MET* gene, known as *MET* proto-oncogene receptor tyrosine kinase, was first identified to induce tumor cell migration, invasion, and proliferation/survival through canonical RAS-CDC42-PAK-Rho kinase, RAS-MAPK, PI3K-AKT-mTOR, and β-catenin signaling pathways, and its driver mutations, such as *MET* gene amplification (*MET*amp) and the exon 14 skipping alterations (*MET*ex14), activate cell transformation, cancer progression, and worse patient prognosis, principally in lung cancer through the overactivation of their own oncogenic and MET parallel signaling pathways. Because of this, *MET* driver alterations have become of interest in lung adenocarcinomas since the FDA approval of target therapies for *MET*amp and *MET*ex14 in 2020. However, after using MET target therapies, tumor cells develop adaptative changes, favoring tumor resistance to drugs, the main current challenge to precision medicine. Here, we review a link between the resistance mechanism and MET signaling pathways, which is not only limited to MET. The resistance impacts MET parallel tyrosine kinase receptors and signals shared hubs. Therefore, this information could be relevant in the patient’s mutational profile evaluation before the first target therapy prescription and follow-up to reduce the risk of drug resistance. However, to develop a resistance mechanism to a MET inhibitor, patients must have access to the drugs. For instance, none of the FDA approved MET inhibitors are registered as such in Chile and other developing countries. Constant cross-feeding between basic and clinical research will thus be required to meet future challenges imposed by the acquired resistance to targeted therapies.

## 1. Introduction

The principal hallmark of tumorigenesis is cell transformation, involving the transition of normal cells into the tumorigenic state, followed by an enhanced cell proliferation, and anchorage-independent growth, which result in cell migration, invasion, and finally metastasis. However, depending on the signaling pathways altered by the driver mutations, the tumor mass would be highly proliferative, invasive, angiogenic, and or metastatic [1]. Assuming that the driver mutations in genes such as *EGFR, ALK, ROS1, HER2*, and *MET* (among others) had demonstrated advantages favoring cell transformation, leading to the expansion of the altered clone, which could be followed by tumor formation and its evolution.

The *MET* gene encodes a member of the receptor tyrosine kinase (RTK) family of proteins, and, since the early 1980s, different authors have studied the effect of *MET* on cancer development, starting by Cooper et al., who were the pioneers in recognizing *MET* gene as a “driver gene” when this concept did not exist yet, describing it as a transforming gene detected in chemically transformed cells [2]. Afterwards, Tward et al. found that the *MET*amp would be able to induce cell transformation and hepatocellular carcinoma (HCC) in mice overexpressing a wild-type allele of human *MET*, although the carcinoma only arose in cooperation with the constitutively active β-catenin expression [3]. Perhaps this was possible through the crosstalk signaling Met/β-catenin since the inactivation of *MET* transgenes induced the regression of HCC [4]. In the same way, Mi et al. speculated whether MET could have initiated tumorigenesis in mice prostates, so they tested this idea designing a conditional Met transgenic mouse that mimicked human prostate cancer through an increased Met expression, which resulted in the oncogenic prostate transformation. Nevertheless, the presence of METamp and *PTEN* deletion leads to prostate neoplasia and prostatic adenocarcinomas, inducing an epithelial-mesenchymal transition and an increase of metastasis events [5]. As a result, METamp caused cell transformation; however, this had to take place in cooperation with another alteration in Met signaling pathways or MET parallel signaling co-activation, which could be needed to support the MET activities. All of this was the initial knowledge about MET in cancer; nevertheless, the development of target therapies against MET was not initiated until the discovery of *MET*ex14, which is by far the most common *MET* mutation. In this review, we will discuss the current therapeutic opportunities for *MET* driver alterations and the current understanding of MET resistance mechanisms.

## 2. MET in Cancer Initiation and Driver Mutations

### 2.1. MET Amplification

In 1996, Ichimura et al. revealed that MET protein and its specific ligand, hepatocyte growth factor (HGF), were highly expressed in lung cancer cell lines, as well as in non-small cell lung cancer (NSCLC) biopsies [6,7]. However, the protein levels did not reveal whether these results were associated with a specific *MET* mutation or with the gene copy number gain (GCN) [8], which could arise from polysomy or amplification. Still, the amplification represents a biologic selection process for *MET* as an oncogenic driver. As *MET*amp has been recognized as a bad prognosis biomarker in NSCLC, HCC, gastric cancer, and triple-negative breast cancer [9,10,11,12], its identification in precision medicine through the GCN is currently calculated using the copy number variation (CNV) from next-generation sequencing (NGS) or using the standard assay fluorescence in situ hybridization (FISH) [13].

The current *METamp* frequency in different solid cancers could be estimated thanks to the AACR Project Genomics Evidence Neoplasia Information Exchange (GENIE), which is an international pan-cancer registry [14]. According to GENIE, *MET*amp represents a 2% in non-small cell lung cancer (NSCLC 10,451 patients), a 1.2% in renal (1556 patients), and hepatobiliary (1854 patients) cancers, followed by a 0.4% in colorectal (7370 patients), 0.2% in ovarian (4481 patients), breast cancer (8365 patients), and prostate cancers (3530 patients). NSCLC has the highest *MET*amp and driver mutations frequency (Table 1), as the *MET*ex14 is the most common MET driver and actionable alterations [15,16], where, according to cancer genomics, a driver mutation starts the tumor development, and an actionable mutation is a driver alteration that has assigned a target therapy. Now, as much as *MET*amp and *MET*ex14 are recognized as actionable because they account for different target therapies replacing the conventional chemotherapies; despite the success in *MET*amp, the therapy response will depend on the GCN degree, as we will later review.

### 2.2. MET Exon 14 Skipping Alterations

The *MET*ex14 was first reported in small cell lung cancer and then in NSCLC patients. Furthermore, when *MET*ex14 was expressed in normal mouse NIH3T3 fibroblasts, cells were transformed and then became tumorigenic in vivo, which confirmed *MET*ex14 as a driver alteration [17], whereas Paik and colleagues were demonstrating METex14 tumor cells were sensitive to MET tyrosine kinase inhibitors (TKIs), and the clinical benefit for NSCLC patients was demonstrated [18].

Normally, in *MET* pre-mRNA, the introns flanking the exon 14 are spliced out, resulting in an mRNA containing exon 14, which encodes the juxtamembrane domain (JMD), which is key for MET protein degradation. The *MET*ex14 causes the loss of JMD when mutations at the splice donor or acceptor sites result in exon 14 loss, such as base substitutions, insertions, deletions, and intronic noncoding regions immediately adjacent to the splice acceptor site and the whole-exon deletion. When some of these alternatives derived in a truncated MET receptor, it shows a constitutive expression because the loss of the Tyr1003 residue located in the JMD prevents the binding of the E3 ubiquitin ligase Cbl and proteasomal degradation, which have shown overactive MET signaling pathways, triggering an exacerbate cell proliferation and invasion, contributing to the evolution of cancer and bad prognosis [19,20].

**Figure 1:**
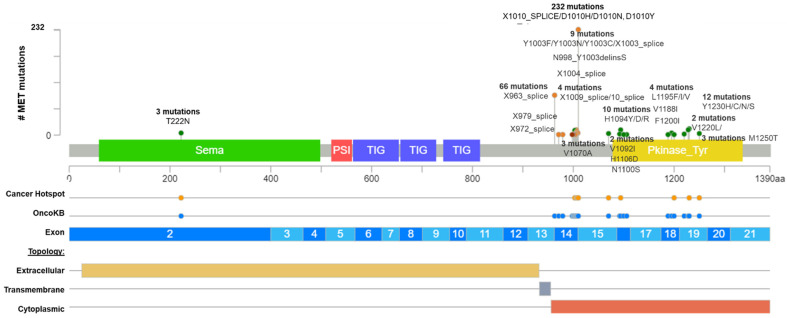
Common *MET* driver mutations impact principally the juxtamembrane and kinase domains (exon 14). Lolliplot of MET protein domains (Sema, PSI, TIG, juxtamembrane, and Pkinase) showing common driver mutations identified in solid tumors from Table 1. Additionally, the protein structure shows the cancer hotspot (yellow circle), OncoKB prediction therapies (blue circles), exons numbered (blue and light blue rectangles), and protein topology. Yellow is the extracellular region, red, the cytoplasmic region, and, gray, the transmembrane region.

*MET*ex14 occurs in 3 to 4% of NSCLC patients (Table 1), and it has been identified as a potent therapeutic target encouraging the approval of TKIs [21,22]. However, the actionability of different variants which originate the *MET*ex14 alteration have not been totally validated yet. Currently, several *MET* mutations are recognized as actionable METex14 by the approved test Foundation One (such as splice site 2888-10_2911del34, splice site 2888-37_2888-30delCGTCTTA, splice site 2888-18_2888-5del14, D1010N, splice site 3028+2T>C, splice site 2999_3028+4del34, splice site 3028+1G>A, and splice site 3028_3028+2delGGT), which were searched in the GENIE public database, and, as a result, only D1010N was found, representing 9.6% of 439 *MET* driver mutations (Figure 1 and Table S1). In addition, other frequent mutations recognized as a driver by GENIE impacting the exon 14 of *MET* were found, as the X1010_splice (29%), followed by the X963_splice (20.9%) and D1010H (9.7%) (Figure 1).

After that, we interrogate all these variants according to the bioinformatic driver predictors, and OncoKB as the actionability predictor, which is a precision oncology knowledge base developed at Memorial Sloan Kettering Cancer Center that contains biologic and oncogenic effects, prognostic, and predictive significance of somatic alterations [23]. As is shown in Table S1, sixteen alterations in exon 14 (affecting 374 patients) were recognized by different predictors, including OncoKB as oncogenic/likely oncogenic mutations with level 1 of evidence, which means these mutations concur with an FDA-approved drug. According to these results, many driver and actionable *MET* mutations had not been functionally or clinically validated yet (Table S1). Additionally, there is an extensive list of mutations in the *MET* hot spot between exons 14 and 19 with drugs predictions, which must be evaluated as actionable alterations (Figure 1 and Table S1).

Still, the validation and approval of additional *MET* driver alterations should be the next step to offer many potentially *METex14* targetable mutations [24,25]. Indeed, a study identified five hundred genetic alterations that lead to *METex14*, and the analysis revealed that the most frequent regions impacted were the splice donor site (42%), followed by the polypyrimidine tract (15%), the splice acceptor site (~5%), and both the splice acceptor site and the polypyrimidine tract (13%). All these alterations resulted in the elimination of exon 14 with an mRNA containing the exon 13 fused to exon 15 [26].

Thereby, given the diversity of alterations leading to *METex14* revealed in mRNA, the diagnosis sensitivity could challenge the identification of them in DNA assays. In contrast, RNA approaches directly identify 13–15 exons fusion in the transcript. For this reason, the amplicon-based approaches may fail to find *METex14* alterations because it does not allow the detection of large deletions. However, hybrid capture is more amenable to detecting the alterations leading to *METex14*. Furthermore, this method generally isolates larger fragments of DNA, including sequences that flank the regions of interest, compared with amplicon-based methods when using DNA as the input material [27]. Additionally, 60% of positive results according to the RNA-based assay were negative using the DNA-based assay [28]. Likewise, the mRNA-based quantitative reverse transcriptase RT-PCR demonstrated 100% sensitivity in detecting METex14, compared to 61.5% sensitivity using conventional DNA-based Sanger sequencing [29], so RNA analysis seems to be best way to identify METex14 to follow with target drugs’ prescriptions.

## 3. Actionable MET Mutations and MET-Target Therapies

The validation of new driver alterations and their actionability helps improve target drug availability, which contributes to the development of precision medicine [30]. As it is conceivable that some cancers are primarily driven by the *MET* mutations [31], to date, *METamp* and *METex14*, have shown their actionability in NSCLC, such as prognosis, and expanding the therapeutic opportunities for patients without EGFR and ALK as usual alterations [32].

The NSCLC subtype adenocarcinoma has been the most advanced cancer subtype in the implementation of targeted therapies, and at the current date, two categories of MET tyrosine kinase inhibitors (TKI) have been validated (Table 2), such as type I, which included crizotinib, capmatinib, tepotinib, and savolitinib that binds to an active MET conformation, and type II (cabozantinib, merestinib, and glesatinib) that binds to an inactive MET [33]. The TKI crizotinib was the first target drug FDA-approved with multi-kinase activity against MET, ALK, and ROS1 [34,35,36]; however, the best patient response to crizotinib was in ALK-positive NSCLC patients compared to MET and ROS1 positive patients [37].

### 3.1. Type I MET-Targeting Drugs Directed to Actionable Mutations

#### 3.1.1. Crizotinib

Crizotinib was validated in 2010 for *METamp* in NSCLC patients [38], and in 2016, its efficacy and safety on METex14 advance NSCLC (aNSCLC) patients were confirmed [39]. However, concerning *METamp*, not all GCN levels experience the same therapeutic results, as was demonstrated in the clinical trial PROFILE-1001 in 2021 (NCT00585195), where low levels (≥1.8–≤2.2 copies), medium (>2.2–<4 copies), and high (>5 copies) experienced a progression-free survival (PFS) of 1.8, 1.9, and 6.7 months, respectively [40]. Drilon et al. [41] reported the safety and response rate of 69 patients with METex14 alteration from an expansion cohort of NCT00585195, reporting an objective response rate of 32%, mPFS of 7.3 months, and OS of 20.5 months (95% CI, 14.3–21.8).

#### 3.1.2. Tepotinib

Tepotinib is a kinase inhibitor that targets MET alterations, including METex14. It was FDA approved in 2021 for patients with metastatic NSCLC (mNSCLC). Tepotinib inhibits the hepatocyte growth factor of dependent and independent MET phosphorylation and MET-dependent downstream signaling pathways [42]. In the VISION trial, 152 METex14 patients were treated with Tepotinib (NCT028649920, Table 2), which was associated with an objective response rate of 56%. The median duration of response and mPFS was 11.1 and 8.5 months, respectively. In patients with central nervous metastasis, the observed response rate was 55%, with mPFS of 10.9 months.

#### 3.1.3. Capmatinib

Capmatinib is a selective MET TKIs ATP-competitive MET inhibitor (type Ib) and was screened against >400 kinases, showing high selectivity for MET. In 2020, capmatinib was FDA approved for patients with mNSCLC harboring METex14 showing a significant antitumor activity, particularly in those not previously treated, and this drug has been approved in several countries including UE, the EU, US, Switzerland, and Japan. The overall response rate observed with capmatinib in patients with METex14 skipping mutation was 41% in 69 patients who had received previous treatment and an ORR of 68% in patients naïve to treatment. In terms of time of response, 82% of patients who had been previously treated with other lines of therapy had tumor response at the first CT scan of evaluation. Capmatinib showed high response rates in patients with intracranial disease with 33% of patients achieving complete response. In addition, the response to *METamp* was higher in tumors with a high GCN than in those with a lower GCN (Table 2). Capmatinib also has been documented in *METamp* in combination with gefitinib (NCT01610336 and NCT01324479) in NSCLC with METex14 and *MET* GCN > 6 (high) [43]. Lower efficacy was observed among NSCLC patients with *MET*ex14 skipping mutations who previously had received one or two lines of therapies, with an overall response in 41% of the patients but showed limited activity in patients who had *MET*amp with a gene copy number of less than 10 (Table 2).

#### 3.1.4. Savolitinib

Savolitinib is a potent and highly selective oral MET TKI that provides clinical benefits for patients with mNSCLC papillary and clear cell renal cell carcinoma (RCC), gastric cancer, and colorectal cancer [44]. In a phase 2 trial that included patients with pulmonary sarcomatoid carcinoma, a rare and poorly differentiated type of NSCLC and NSCLC adenocarcinoma treated with savolitinib showed a similar objective response rate of 42%. Most of the patients were male and the median age of the 70 patients was 68.7 years. The median time to response was 1.4 months with a high disease control rate (82%). The pulmonary sarcomatoid carcinoma median duration of response was 17.9 months, suggesting that the treatment response to savolitinib was acceptable, durable, and rapid in patients with pulmonary sarcomatoid carcinoma and other NSCLC subtypes positive for METex14 [45]. This drug had been approved China’s National Medical Products Administration (NMPA) (conditional on the results of a phase III trial) to treat mNSCLC with METex14 in patients who have progressed after or who are unable to tolerate platinum-based chemotherapy. Currently, the FDA has not approved savolitinib against METex14 yet. However, it was FDA approved mixing savolitinib with osimertinib therapy because it helps patients with the EGFR-mutant that had developed resistance to prior EGFR-targeted therapies through MET-gene amplification [46].

In relationship with the immunotherapy, *MET* alterations have gained relevance since NSCLC patients with low *MET* GCN (*METamp* GCN ≤ 10) have been treated with immune checkpoint inhibitors (ICI), which showed a median survival of 19 versus 8 months with chemotherapy. Nevertheless, in patients with high amplification levels (GCN ≥10), the median survival was 36 months with ICI compared to 4 months with chemotherapy. However, the patients with METex14 did not present significant differences, and there was a trend toward improved survival with ICI therapy compared to chemotherapy (16.0 months versus 10.0 months) [21].

### 3.2. MET Alterations and Treatment in the Context of Mestastasic NSCLC

In general, the treatment for mNSCLC, which occurs in one-third of METex14-altered lung cancers at diagnosis [47], currently is based on systemic therapy using cytotoxic and/or molecularly targeted agents and palliative radiotherapy for symptomatic metastases with poor prognosis. Patients with METex14–positive NSCLC and brain metastasis have been through a long journey to find the target drug with good central nervous system activity [48,49]. Since 2020, the FDA has approved three target drugs for mNSCLC patients with METex14 alteration, such as capmatinib, which showed an intracranial response in 7 of 13 patients (54%) [43]. The intracranial responses were also observed in a phase II trial of savolitinib and the VISION phase II study of tepotinib that had 11 patients with brain metastases and a response rate of 55%, with a median duration of response of 9.5 months, and median duration of progression-free survival of 10.9 months [42]. Furthermore, on 10 August 2022, the FDA granted regular approval to capmatinib for adult patients with metastatic non-small cell lung cancer (NSCLC) whose tumors have a mutation leading to METex14.

The *MET*amp shows a vast heterogeneity in GCN; however, the best therapy prognosis has been seen in the high levels (GCN > 10) as depicted in Table 2, where the therapy success decreases proportionally to GCN decrease. Therefore, if the *MET*amp is increased without any known actionable alteration that drives the tumor, the evaluation of immunotherapy additional to MET TKIs is recommended. As such, the MET inhibitors plus immune checkpoint inhibitors (ICIs) strategy has been improving pancreatic cancer immunotherapeutic efficacy [50], evidencing potential crosstalk between c-MET inhibition and immune escape since PD-L1 expression positively correlates with METamp [51]. Furthermore, a clinical trial in progress is studying the safety and efficacy of capmatinib plus pembrolizumab vs. pembrolizumab alone in NSCLC with PD-L1 ≥ 50% (NCT04139317), which might be the confirmation of a new improvement to the only MET-TKI therapies in patients with *MET* alterations.

Additionally, an mNSCLC case report treated with chemotherapy switched to pembrolizumab (PD-L1 70%) with tepotinib that enabled stable disease following this therapeutic decision for 31 months [52]. Accordingly, the MET alterations and high PD-L1 expression must be considered recurrent with actionable co-alterations in NSCLC with the option to combine MET-TKIs with ICIs, which could also be the best strategy in case of resistance to other target therapies. Nonetheless, the evidence to support clinical recommendations, such as the best sequentially of MET inhibitors and immunotherapy, is still limited. In addition, considering the advance stage patient setting, therapy options are limited by drug toxicities, patient comorbidities, and adverse reactions [42].

## 4. Resistance Mutations as a Bypass of MET-Target Therapies

Even though targeted therapies have increased the life expectancy of patients with druggable driver alterations [53], their efficacy could be limited when tumor cells acquiree therapy resistance mutations, which, depending on the resistance mechanism used, could impact the same molecular target (On-target), MET parallel tyrosine kinase signaling, or its own signaling pathways with the aim of treatment breakdown [54,55], so, the knowledge about the driver’s signaling pathways alterations will be relevant to predicting the possible drug bypasses at diagnosis [56].

Although the study of signaling pathways has been confined to basic science, it gives insight into relevant information for precision medicine when the driver alterations are spatially localized in their signaling pathways to predict future resistance mechanisms according to a personalized somatic mutational profile analysis, which does not only consider the diagnosis of actionable mutations with the highest mutation frequency, because intratumor low-frequency alterations should also be considered as a possible future resistance mutation. The manual curation of somatic mutations after the NGS bioinformatic analysis is rich in information to envisage possible sensitivity and resistance alterations when considering known variants at low frequency could then be stimulated by the target drugs based on the sub clonal evolution, where the treatments may serve as a selective pressure to increase the frequency of these possible resistance mutations [57].

### 4.1. Acquisition of On-Target Mutations Resistance to MET TKIs

**Figure 2:**
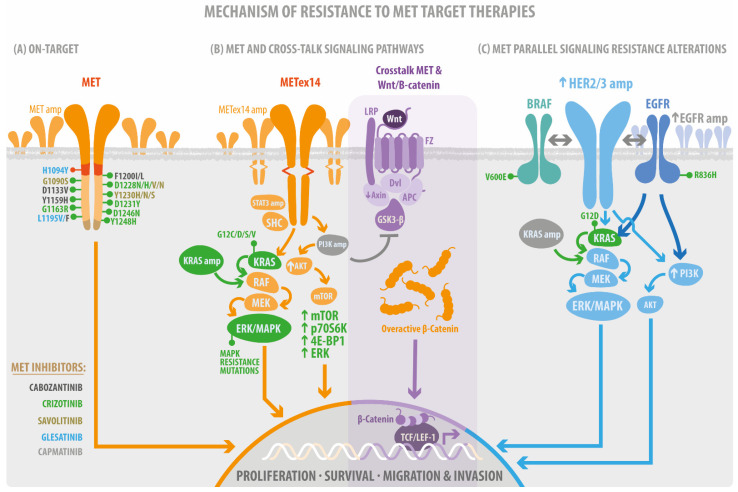
Mechanism of resistance to MET target therapies. (**A**) the on-target resistance mutations. These mutations impacted the Juxtamembrane domain (RED) and the Tyrosine kinase domain (orange). The multi-docking site (brown) without evidence of resistance mutations. (**B**) Canonical MET signaling pathways acquire resistance mutations such as STAT, PI3K, and KRAS amplification (amp), AKT, mTOR signaling overexpression (arrow up), KRAS mutations, and crosstalk’s mediate PI3Kamp and GSK3b inhibition, Axin downregulation (down arrow) followed by overactivation of b-catenin signaling. (**C**) The acquired resistance mutations impact the MET parallel receptor tyrosine kinase signaling after using MET-TKI. Therefore, the colors of the target drugs (MET inhibitors) are related to the color of mutations.

The scene of resistance mechanisms to MET TKIs has not been well characterized, and its frequency impact is unknown. However, acquiring resistance mutations could have on-target preference after MET-TKIs fail (Figure 2A). One form to identify resistance mutations is through patients’ analysis before and after MET-TKIs therapies.

A study considering 20 plasma and tissues analyzed by NGS from *MET*ex14 NSCLC patients demonstrated known and suspected resistance mechanisms in 15 patients. The on-target acquired resistance mechanisms, including MET kinase domain mutations in codons H1094, G1163, L1195, D1228, and Y1230 in seven patients (35%), showed high *MET* exon 14-mutant allele amplification [33]. Another group of patients with METex14 mutations demonstrated specific resistance mechanisms to crizotinib and type II MET TKI glesatinib. In four cases (33%), acquired MET alterations were identified, including one patient with amplification of the mutated METex14 allele and three cases with MET tyrosine kinase domain secondary site mutations; in two of these cases, more than one MET resistance mutation was present in the same patient. Secondary mutations in MET included H1094Y, G1163R, L1195F, L1195V, D1228N, Y1230H, and Y1230S [58]. In addition, it has been reported that MET D1228 and Y1230 are the hotspots for the secondary resistant mutation for type I MET TKIs in NSCLC carrying *MET*ex14 using in vitro models (Figure 2A) [59,60]. After the use of the TKI gefitinib, 12 patients with advanced NSCLC were analyzed using capture-based targeted ultra-deep sequencing, revealing MET Y1248H and D1246N as acquired mutations in two patients, further confirming their resistance against type I MET-TKIs in silico, in vitro, and in vivo, which could be solved using TKIs type II, according to in vitro experiments’ results [61].

### 4.2. MET Parallel Signaling Resistance Alteration

MET activation is an effective resistance mechanism to targeted therapies against several RTKs, including *EGFR, HER2, VEGFR*, and against signaling hubs such as BRAF [62]. An example is *MET*amp, which has been shown to be one of the most relevant mechanisms responsible for the acquired resistance against EGFR TKIs for both first and second-generation TKIs. It has also been observed as the most common resistance mechanism affecting 15–20% of ctDNAs in patients with NSCLC (NCT02296125).

The concomitant use of MET inhibitors, such as crizotinib, in combination with osimertinib, can improve the sensitivity of patients with osimertinib-resistant EGFR mutations and with amplified MET, which were detected using ctDNA [63]. Currently, the combination of nazartinib with capmatinib is under study (NCT02335944), and tepotinib has been tested in combination with gefitinib in participants with T790M negative *MET*amp [64] positive in locally advanced or mNSCLC with *EGFR* mutations, and the results do not show significant improvement in OS compared to standard chemotherapy; one reason for this response is that this combination may have better response rates in high MET overexpression (IHC3+) or METamp (GCN ≥ 5).

*METamp* due to gefitinib resistance drives the drug bypass through ERBB3 dependent activation of PI3K [65], and *METamp* cell lines treated with capmatinib have suggested that the activation of EGFR-PIK3CA signaling may mediate the resistance without any *EGFR* activating mutation or amplification (Figure 2C). Therefore, it may indicate a preferable PI3K signaling pathway activation, independent of *MET* or *EGFR* acquiring resistance or primary alteration [66]. Perhaps, the combination of capmatinib with gefitinib is a promising treatment for patients with EGFR-mutated and *METamp* in NSCLC [67]. Acquired amplification of wild-type *EGFR, KRAS, HER3*, and *BRAF* has been reported and was detected in 45% of *MET* exon 14-mutant NSCLC patients previously treated with crizotinib [33].

### 4.3. Resistance Mutation on MET Signaling Pathways

As we noticed in the beginning of this review, the *MET* gene is a critical driver. In addition, the signaling pathways affected by *MET* alterations are essential to predicting the tumor phenotype, as it is a proliferative or invasive disease. The knowledge of the signaling pathways impacted by the driver mutations is becoming more relevant following the initial studies of the resistance mechanism to target therapies. Still, the design of specific target therapies directed to driver mutation related to specific altered signaling pathways has not been accomplished in oncology. Until now, the MET signaling pathways are affected by resistance mutations after Met-TKIs, and, in some cases, co-alterations in the same signaling pathways have been reported (Figure 2B). So far, the solution has been to test the combination of target therapies.

#### 4.3.1. MET/RAS/MAPK, the Proliferative and Survival Signaling

MET activates RAS through the recruitment of effectors [68], which subsequently recruit Sos that convert inactive RAS into an active conformation (RasGDP RasGTP) [69]. RasGTP successively activates Raf, Mek, MAPKs, ERK, JNK (Jun N-terminal kinase), and p38 (HOG). When MAPKs signaling enters the nucleus, it activates transcription factors such as Elk1, Etsl, [70], and c-Myc [71], promoting cell proliferation and cell cycle progression [72]. MAPK can also promote tumor cell invasion, which is early coordinated by HGF-SC/MET [73,74], as depicted in Figure 2B.

Prominent co-occurring RAS–MAPK pathway gene alterations were detected in aNSCLCs patients with *MET*ex14 alterations compared with EGFR, demonstrating an association between decreased MET treatment response and the RAS-MAPK pathway co-occurring alteration [75]. Enrichment analysis of transcriptomic data from patients with METex14 revealed the preferential activation of the KRAS (G12S) pathway in lung cancers as a crizotinib-resistant response [76].

KRAS activation also mediates resistance to targeted therapy in METex14 NSCLC and tumor cell persistence through MAPK signaling overactivation [77]. This similarly happens with the selective Met inhibitor tepotinib, even with its prolonged on-target activity [42]. In addition, Rotow et al. demonstrated a resistance overcome when combining crizotinib and the MEK inhibitor trametinib polytherapy in METex14-mutated preclinical models. However, the early evidence of molecular response to combined crizotinib and trametinib therapies in a patient with acquired KRAS amplification was poorly tolerated in the context of overall clinical decline, with fatigue, fluid retention, and diarrhea [75,76].

#### 4.3.2. MET/PI3K/AKT/mTOR Cellular Motility and Invasion Signaling

This signaling pathway is activated by the HGF/SF receptor and includes the generation of D-3-phosphorylated inositol phospholipids (Figure 2B), which regulate cytoskeletal functions, membrane trafficking, and receptor signaling by recruiting protein complexes to cell- and endosomal-membrane, as demonstrated early in 1991 [78]. PI3K pathway alterations are also common in *METex14* NSCLC and confer primary resistance to *MET* TKIs. Accordingly, by combining a *MET* TKI with a PI3K inhibitor, the sensitivity to MET TKIs is restored in preclinical models [79]. *MET* has also been described as a potential therapeutic target for multiple myeloma (MM), where Akt/mTOR is a crucial signaling component through which Met protects multiple myeloma cells from chemotherapy-induced growth inhibition and apoptosis. c-Met/Akt/mTOR pathway is a potential therapeutic target to overcome the chemoresistance of MM [80]. In fact, c-Met depletion significantly enhanced the sensitivity of U266 cells to bortezomib in vitro, indicating that c-Met promotes chemoresistance in MM. Additionally, the knock-down of Met enhanced the cytotoxicity and caspase-mediated apoptosis in human MM U266 cells after bortezomib treatment. These findings raise the possibility that novel combined pharmacologic inhibitors of the c-Met⁄Akt⁄mTOR pathway could enhance the effectiveness of bortezomib in the treatment of myeloma [80].

A study investigated the therapeutic potential of cabozantinib using a large panel of HCC cell lines, and distinct oncogene-driven HCC in mice, where cabozantinib inhibited c-MET and its MAPK signaling activity; however, it was ineffective in inhibiting the MET/Akt/mTOR cascade, but mixing cabozantinib and pan-mTOR inhibitors, has shown synergistic properties in inhibiting HCC growth in vitro and in driving tumor regression in mice [81].

#### 4.3.3. MET/Src/Fak Mechanosensing Signaling

Focal adhesion kinase (FAK) regulates the reorganization of the actin cytoskeleton, cell polarization, cell migration, and adhesion, spreading through MET in medulloblastoma [82]. The combined targeting against MET and FAK could be advantageous for medulloblastoma therapy [82], and the interaction of FAK with MET was required for HGF-induced cell invasion [83], whereas Src-dependent phosphorylation of MET requires cell-matrix adhesion, as well as actin stress fiber assembly. Phosphorylation of FAK by Src is also required for Src-induced MET phosphorylation. Thus, a novel role of the Src/FAK interaction network in the positive regulation of MET activation occurs in an HGF-independent manner in breast epithelial cells and neuronal cells [84].

The inhibition of MET alone has been demonstrated to have limited efficacy in colon cancer; although the Src inhibitor, dasatinib, reduced MET autophosphorylation and decreased MET phosphorylation by stimulating factors, the inhibition is not complete. In addition, the simultaneous inhibition of MET and Src demonstrated that the combination treatment reduced cell viability and increased the apoptosis rate in mutant and wild-type Ras colon cancer cells [85].

In recent years, it has been reported that activation of the MET/FAK signaling axis leads to CDK4/6-independent CDK2 activation, and the blockage of the three targets reduces cell proliferation and enhances tumor growth inhibition in vivo [57]. In addition, verticillin, targeting MET/FAK/Src, inhibited the migration ability through HGF/MET deactivation in gastric and cervical cancer cells [86].

A series of studies confirmed the importance of FAK/Src signaling in resistance to first-(erlotinib), second- (afatinib), and third-generation (osimertinib) EGFR TKIs; thus, with the current diagnosis and mono target therapies against MET, this signaling could be implicated in MET-TKI resistance with an emphasis in metastatic tumors because it activates the signaling pathways such as MET/FAK involved in polymerization and contraction of the actin cytoskeleton to cell migration, followed by intra-extravasation through invasion of basal membrane and metastasis.

Crosstalk among signaling in HCC, such as the MET/β-Catenin axis, resulting in FAK is required for MET/β-Catenin-driven hepatocarcinogenesis. Additionally, in this study, the authors demonstrated that FAK deficiency in hepatocytes largely blocks HCC development induced by MET/β-catenin [34,35].

#### 4.3.4. MET/Wnt/β-Catenin

The crosstalk between HGF/Met and Wnt/β-catenin is strongly implicated in hepatocarcinogenesis [83,87]. It has also been observed as active in glioblastoma, the most lethal and common type of primary brain tumor. The axis MET/Wnt/β-catenin is critical to maintaining cancer stem cells, which possess high levels of MET and Wnt/β-catenin to drive tumor propagation [88]. In the same line, in HCC, targeting Met with tepotinib alone had a minor effect on Met-β-catenin-HCC development, although tepotinib improved overall survival by 1.5–2 weeks. Thus, single therapy with the Met inhibitor will be insufficient for sustained response in Met/β-catenin HCC, requiring assessment of additional combinations [89].

A mechanism of c-Met inhibitor resistance in melanoma was suggested using melanoma in vivo and in vitro models with high levels of phospho Met. After inducing the resistance to MET-TKI (SU11274 and JNJ38877605), cell lines displayed upregulation of phosphorylated Met and overactive β-catenin, suggesting the involvement of the Wnt pathway (Figure 2B). When Met activates Akt, it then inhibits GSK-3 and or directly phosphorylates β-catenin at Ser552, which enhances β-catenin nuclear accumulation [90]. Interestingly, the resistance mechanism was not mediated here by mutations in the Met tyrosine kinase domain (TKD) [91].

Finally, we used all resistance mutations from Figure 2 to investigate co-mutations related to resistance, showing that most patients have only one driver mutation. Still, GENIE samples showed co-mutations in MET and MET with molecules as part of its parallel signaling pathways (Figures S1 and S2). However, information about WNT1 is unavailable because this gene is not part of the GENIE sequencing panel. For this reason, if we consider resistance mutation since the initial diagnosis, it would be relevant to analyze mutations related to MET signaling pathways to predict or avoid all known resistance mutations associated with MET target therapies.

## 5. Conclusions

A complete understanding of the driver mutations and pathways altered is essential to identify potential therapeutic options and vulnerabilities to the therapies, such as treatment resistance. The relevance of a deep analysis of tumor mutational profile in a study of METamp as a consequence of EGFR TKIs resistance therapies detected clones displaying METamp at a shallow frequency before any treatment with an EGFR TKI, suggesting that these clones were selected under therapeutic pressure [92]. Perhaps, in an initial diagnosis, the prediction of potential resistance mutations will consider analyzing the low-frequency alterations related to driver and/or actionable mutations’ signaling pathways. Nevertheless, to develop treatment resistance, patient access to drugs is a must. Currently, in Chile, none of the drugs approved by the NMPA, FDA and EMA against MET actionable alteration are registered [93], so patients whose tumors harbor actionable variants in the *MET* gene are facing a significant disparity in the access to target therapies. In Chile, comprehensive tumor profiling is not part of the lung cancer standard of care, or any national health policies. Precision medicine, and target drugs’ implementation is challenging due to the high cost and lack of biomarker testing options. Patients diagnosed with NSCLC should undergo examination for EGFR, ALK, ROS1, and PDL-1 protein expression according to the drugs registered and available in Chile. Therefore, MET mutations are not considered in the evaluation, and there are no registered MET inhibitors in Chile, except for crizotinib, but it is approved only for patients with ALK and ROS1 alterations. If a lung cancer patient in Chile is diagnosed with a MET actionable alteration, off label use of crizotinib or participation in MET specific inhibitor clinical trial are possibilities. Standard of care treatment option would include surgery, chemotherapy, radiotherapy, or palliative care for these patients. As of today, there is not any public indication of any attempt to register MET inhibitors in Chile. Expectations regarding comprehensive tumor profiling and better access to high-cost drugs resurged with the implementation of recently approved legislation (a Chile National Cancer Law).

## Figures and Tables

**Table 1:** METex14 and METamp frequency in NSCLC, renal, hepatobiliary, colorectal, ovarian, breast, and prostate cancers in the GENIE cohort [13]. Germinal mutations, uncertain significance variants, and patients missing MET analysis were filtered out using available tools at https://genie.cbioportal.org/ (last time accessed on 10 November 2022).

Cancer Type	N° GENIE Samples	N° Samples after Filters	METex14	MET Amp
NSCLC	17,137	10,231	4%	2%
Renal	1986	1556	1.2%	1.2%
Hepatobiliary	2517	1854	0.4%	1.2%
Colorectal	11,893	7370	0%	0.4%
Ovarian	4606	4481	0.1%	0.2%
Breast	13,388	8365	0%	0.2%
Prostate	4379	3530	0.1%	0.1%
Total analysis	56,682	36,095	5.8%	5.3%

**Table 2:** Classification of current MET target therapies, and clinical trial results, approved by FDA.

Drug’s Type	Drug Name	Clinical Trial	Alterations	ORR	DOR	PFS	Disease Control Rate
					(Months)	(Months)	
**Selective type Ia**	Crizotinib	PROFILE-1001	METex14	32%	9.1	7.3	
Multitarget TKIs		(NCT00585195)	GCN ≥1.8–≤ 2.2	33%	12.1	1.8	
			GCN ≥2.2–≤ 4	14.3%	3.7	1.9	
			GCN > 4	40%	5.5	6.7	
**Selective type Ib** monotarget MET TKIs	Capmatinib	GEOMETRY mono-1 trial (NCT02414139)	METex14	40.6% previously treated	9.7	5.4	78%
				67.9% treatment-naive	12.6	12.4	96%
		NCT01324479	MET GCN > 6	47%	N.S	9.3	80%
	Tepotinib	VISION trial (NCT02864992)	METex14	46%	11.1	8.5	65.70%
	Savolitinib	(NCT02897479)	METex14	49.2%	-	6.9 months	93.40%

## Data Availability

https://genie.cbioportal.org/ (last time accessed on 10 November 2022).

## Supplementary Materials

The following supporting information can be downloaded at: https://www.mdpi.com/article/10.3390/ijms232213898/s1.
