# Supplementary material for: MET Signaling Pathways, Resistance Mechanisms, and Opportunities for Target Therapies
Source: Int J Mol Sci. 2022 Nov 11;23(22):13898. doi: 10.3390/ijms232213898 (PMC9697723; doi:10.3390/ijms232213898)
Supplement: Supplementary file 1 [file ijms-23-13898-s001.zip › ijms-1981646-supplementary.pdf]

Supplementary Figure S1. Oncoplot for *MET* on target co-mutations: Each column represents one lung cancer patient. From GENIE Cohort v11.0-public. User-defined Patient List (17056 patients / 19237 lung cancer samples) - MET alterations.

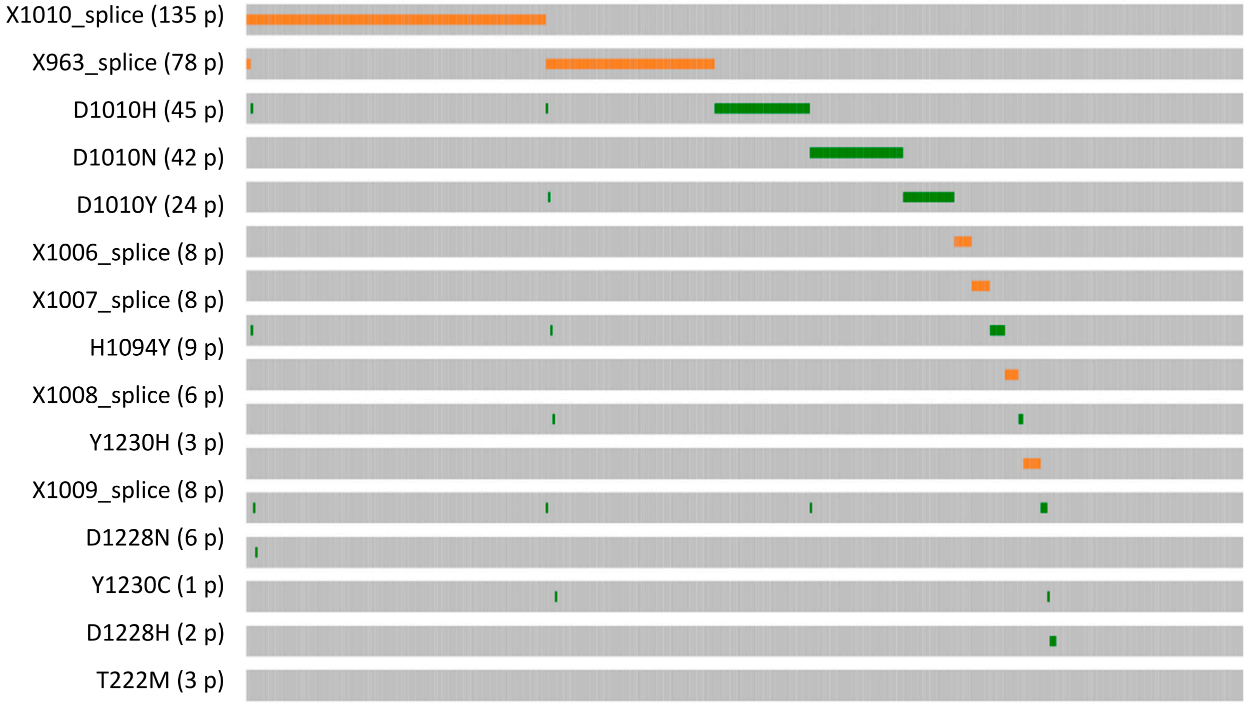

Supplementary Figure S2. Oncoplot for *MET* co-mutations in parallel signaling MET pathways: See the columns. GENIE Cohort v11.0-public. User-defined Patient List (17137 patients / 19319 samples) - MET, KRAS & 11 other genes.

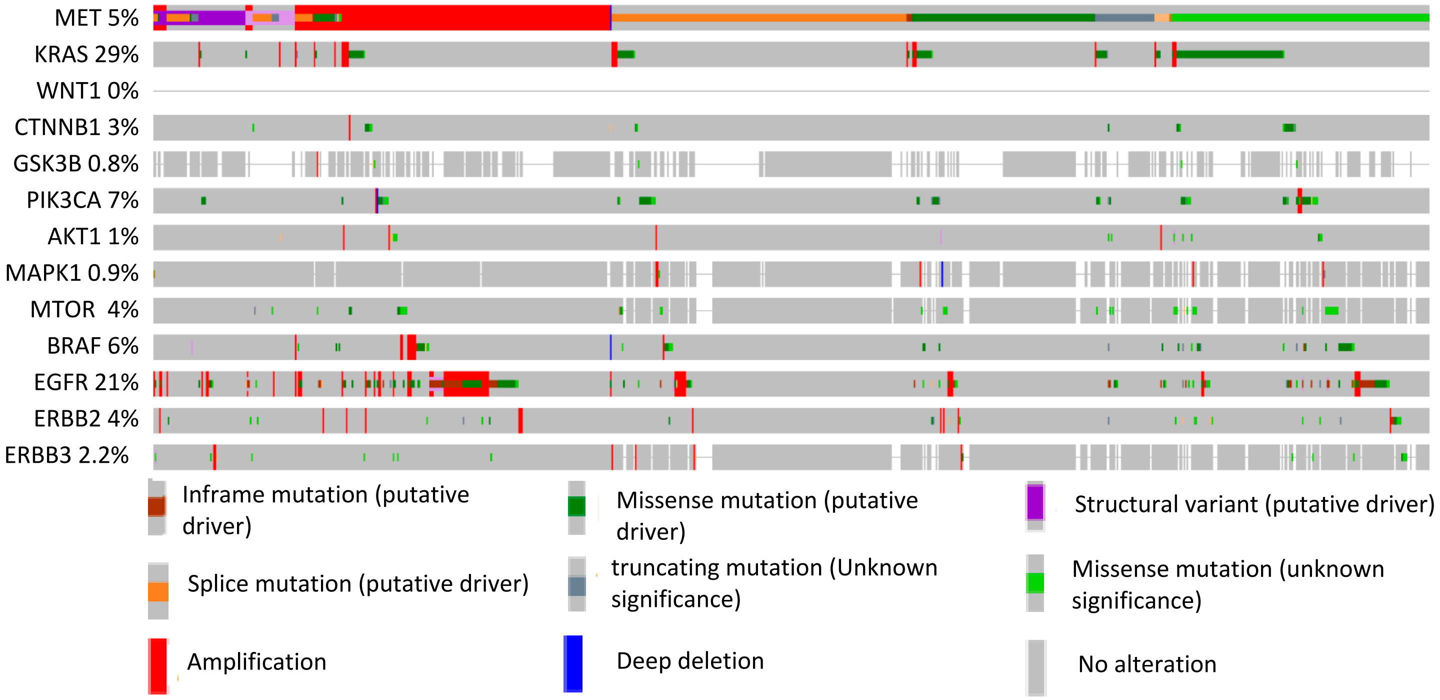

Supplementary Table S1. Driver and actionable statement prediction of known MET variants. All variants showed are predicted driver and actionable. D: Deletereous, T= Tolerated

N°	Protein Change	Variant_ Type	Ex on	Cadd 13	Sif t	Mut Tast er	Fath m Mkl	Lrt	M-Cap	Met alr	Poly phen 2	Driver Statement	Oncogenic Statement	Oncokb´s Evidence Levels
128	X1010 splice	Splice Site	14	25.9	.	D	D	.	.	.	.	predicted driver tier 1	Likely Oncogenic	Level 1
92	X963 splice	Splice Site	14	42	.	D	D	.	.	.	.	predicted driver tier 1	Likely Oncogenic	Level 1
43	D1010H	Missense	14	25	D	D	D	D	D	T	D	predicted driver tier 1	Likely Oncogenic	Level 1
42	D1010N	Missense	14	25.9	T	D	D	D	D	T	D	predicted driver tier 1	Likely Oncogenic	Level 1
28	D1010Y	Missense	14	28.1	D	D	D	D	D	T	D	predicted driver tier 1	Likely Oncogenic	Level 1
8	H1094Y	Missense	16	29.0	D	D	D	D	D	T	D	known in renal carcinoma	Oncogenic	unknown
8	X1006 splice	Frame_ Shift Del	14	27.7	.	D	D	.	.	.	.	predicted driver tier 1	Likely Oncogenic	Level 1
8	X1007 splice	Frame_ Shift Del	14	31	.	D	D	.	.	.	.	predicted driver tier 1	Likely Oncogenic	Level 1
7	Y1230H	Missense	19	48	D	D	D	D	D	D	D	known in renal carcinoma	Oncogenic	Level R2
7	X1008 splice	Frame_ Shift Del	14	26.7	.	D	D	.	.	.	.	predicted driver tier 1	Likely Oncogenic	Level 1
5	D1228N	Missense	19	31	D	A	D	D	D	D	D	predicted driver tier 1	Likely Oncogenic	Level R2
5	X1009 splice	In Frame Del	14	29.6	.	D	D	.	.	.	.	predicted driver tier 1	Likely Oncogenic	Level 1
4	D1228H	Missense	19	27.7	D	D	D	D	D	D	D	predicted driver tier 1	likely oncogenic	unknown
4	T222M	Missense	2	29	D	D	D	D	D	T	D	predicted passenger	Unknown	unknown
4	Y1230C	Missense	19	28.7	D	A	D	D	D	D	D	known in renal carcinoma	Likely Oncogenic	Level 1
3	Y1003N	Missense	14	25.4	D	D	D	D	D	T	D	predicted driver tier 1	Likely oncogenic	unknown
3	V1070A	Missense	15	32	D	D	D	D	D	D	D	predicted driver tier 1	Likely Oncogenic	unknown
3	M1250T	Missense	19	31	D	D	D	D	D	T	D	known in renal carcinoma	Oncogenic	Level 1
3	Y1230N	Missense	19	26.5	D	D	D	D	D	D	D	predicted driver tier 1	Likely Oncogenic	Level 1
3	Y1003F	Missense	14	27.7	D	D	D	D	D	T	D	predicted driver tier 1	Oncogenic	Level 1
2	V1220L	Missense	19	27.5	D	D	D	D	D	T	D	predicted driver tier 1	Unknown	unknown
2	V1092I	Missense	16	31	D	D	D	D	D	T	D	known in CANCER-PR	Oncogenic	level 1

2	D1002G	Missense	14	25.0	D	D	D	D	D	T	P	predicted driver tier 1	Likely Oncogenic	level 1
2	Y1003C	Missense	14	28.5	D	D	D	D	D	T	D	predicted driver tier 1	Likely Oncogenic	Level 1
2	L1195F	Missense	18	31	D	D	D	D	D	D	D	predicted driver tier 1	Unknown	Level 1
1	H1094R	Missense	16	48	D	A	D	D	D	T	D	known in renal carcinoma	Oncogenic	unknown
1	L1195V	Missense	18	27.7	D	D	D	D	D	D	D	predicted driver tier 1	Oncogenic	unknown
1	V1220I	Missense	19	28.2	D	A	D	D	D	T	D	predicted driver tier 1	Likely Oncogenic	unknown
1	Y1230S	Missense	19	26.8	D	D	D	D	D	D	D	predicted driver tier 1	Likely Oncogenic	unknown
1	N998 Y1003delinsS	In Frame Del	14	48	.	D	D	.	.	.	.	predicted driver tier 1	Unknown	unknown
1	H1094D	Missense	16	26.8	D	D	D	D	D	D	D	predicted driver tier 1	Unknown	unknown
1	V1188I	Missense	18	29.1	D	D	D	D	D	T	D	predicted driver tier 1	Unknown	unknown
1	L1195I	Missense	18	48	D	D	D	D	D	D	D	predicted driver tier 1	unknown	unknown
1	X972 splice	Frame_ Shift Del	14	24.9	.	D	D	.	.	.	.	predicted passenger	unknown	unknown
1	X979 splice	Frame Shift Del	14	29.3	.	D	D	.	.	.	.	predicted passenger	unknown	unknown
1	X1001 splice	In Frame Del	14	48	.	D	D	.	.	.	.	predicted passenger	unknown	unknown
1	X1003_splice	Frame_ Shift Del	14	48	.	D	D	.	.	.	.	predicted passenger	unknown	unknown
1	X1004 splice	Frame Shift Del	14	24.9	.	D	D	.	.	.	.	predicted passenger	unknown	unknown
1	T1006 D1010delinsN	In Frame Del	14	48	.	D	D	.	.	.	.	predicted passenger	unknown	unknown
1	F100 D1010delinsY	In Frame Del	14	48	.	D	D	.	.	.	.	predicted driver tier 1	likely oncogenic	unknown
1	N1100S	Missense	16	48	T	N	N	N	T	T	B	predicted passenger	unknown	unknown
1	D1228V	Missense	19	26.7	D	D	D	D	D	D	D	known in LUAD	Likely oncogenic	unknown
1	H1106D	Missense	16	48	D	D	D	D	D	T	D	predicted driver tier 1	Likely Oncogenic	Level R2
1	F1200I	Missense	18	32	D	D	D	D	D	T	D	predicted driver tier 1	Oncogenic	Level R2
1	V1001 F1007del	In Frame Del	14	25.9	.	D	D	.	.	.	.	predicted driver tier 1	Likely Oncogenic	Level 1
1	D1002 F1007del	In Frame Del	14	41	.	D	D	.	.	.	.	predicted driver tier 1	Likely Oncogenic	Level 1
